# Investigation of the Effects of Phenolic Extracts Obtained from Agro-Industrial Food Wastes on Gelatin Modification

**DOI:** 10.1021/acsomega.4c00690

**Published:** 2024-04-24

**Authors:** Huseyin Demircan, Rasim A. Oral, Omer S. Toker, Ibrahim Palabiyik

**Affiliations:** † Faculty of Engineering and Natural Science, Department of Food Engineering, Bursa Technical University, 16310 Bursa, Turkey; ‡ Faculty of Chemical and Metallurgical Engineering, Department of Food Engineering, Yildiz Technical University, 34210 Istanbul, Turkey; § Faculty of Agriculture, Department of Food Engineering, Tekirdağ Namık Kemal University, 59030 Tekirdağ, Turkey

## Abstract

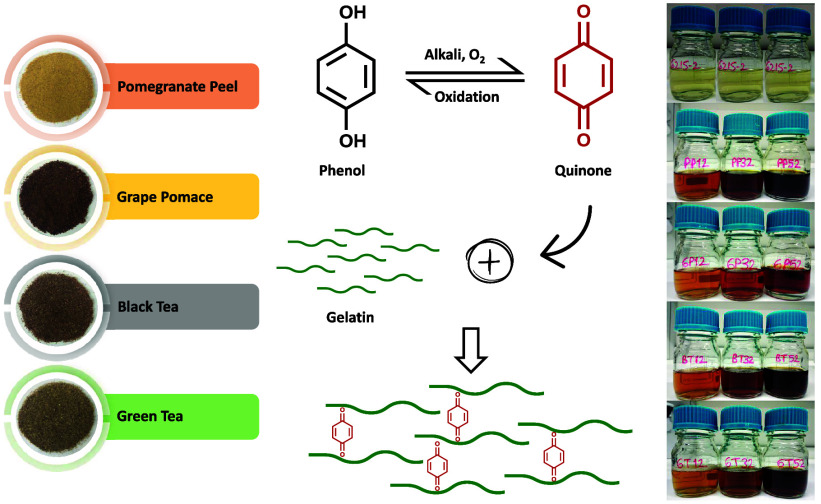

In this study, modified bovine gelatin was produced using the alkaline technique with four different oxidized agro-industrial food waste (pomegranate peel (PP), grape pomace and seed (GP), black tea (BT), and green tea (GT)) phenolic extracts (AFWEs) at three different concentrations (1, 3, and 5% based on dry gelatin). The effect of waste type and concentration on the textural, rheological, emulsifying, foaming, swelling, and color properties of gelatin, as well as its total phenolic content and antioxidant activity, was investigated. Significant improvement in gel strength, thermal stability, and gelation rate of gelatin was achieved by modification with oxidized agro-industrial waste extracts. Compared to the control sample, 46.24% higher bloom strength in the GT5 sample, 5.29 and 6.01 °C higher gelling and melting temperatures in the PP5 sample, respectively, and 85.70% lower *t*_model_ value in the GT3 sample were observed. Additionally, the total phenolic content, antioxidant activity, foam, and emulsion properties of the modified gels increased significantly. This study revealed that gelatins with improved technological and functional properties can be produced by using oxidized phenolic extracts obtained from agricultural industrial food wastes as cross-linking agents in the modification of gelatin.

## 1 Introduction

Gelatin is a water-soluble fibrous protein obtained by thermal denaturation or partial hydrolysis from animal collagen found in skin, bone, and tendon.^1,2^ It is widely used in the food, pharmaceutical, cosmetics, materials, and photography industries due to its plasticity, stickiness, and low antigenicity.^1,3^ Gelatin also has some important functional properties such as gelling, film-forming, emulsifying, foaming, and swelling and is used as an emulsifier, foaming agent, thickener, clarifier, stabilizer, scaffold material, drug carrier, food preservation film/coating, etc.^4−9^ However, the mechanical and thermal properties of gelatin limit its potential use in some areas.^3,10^ Various techniques have been investigated for their potential to improve the functional properties of gelatin.^11^ The chemical modification of gelatin involves the use of compounds such as genipin,^1,3,12^ carbodiimides,^1^ glyoxal,^12^ dialdehyde carboxymethyl cellulose^13^ and other aldehyde-containing substances,^3,10,12^ sodium alginate,^14^ low acyl gellan,^15^ κ-carrageenan,^16^ oxidized microcrystalline cellulose,^17^ and oxidized corn starch-based nonionic biopolymers.^5^ Physical methods such as ultraviolet irradiation,^3,10^ γ irradiation,^1^ and high-pressure^1,18^ techniques are also used in gelatin modification. In the enzymatic modification, transglutaminase^1,10,12,18^ is more commonly used. The selection of modification techniques and materials is limited by various factors such as inadequate enhancement of gelatin’s functional properties, formation of heterogeneous gels, challenges in application, potential toxicity of chemicals, and high costs.^3,10,12^ Therefore, it is important to use efficient techniques and cross-linking agents that are suitable for use in foods, nontoxic, cheap, and easily available.^3,10^

Phenolic compounds, abundant in plants, are the main type of secondary metabolites and exhibit a broad range of structures and functions. They usually have an aromatic ring with one or more hydroxyl substituents. Polyphenols can react with polypeptides through noncovalent and covalent interactions.^19^ Under alkaline conditions, the diphenol part of polyphenols is oxidized by molecular oxygen to quinone, which can interact with the amino or sulfhydryl side chains of proteins. Polyphenols can also interact hydrophobically, electrostatically, and by hydrogen bonding with charged proteins.^11,20^ These interactions enable polyphenols^10,12,21,22^ or polyphenol-rich plant extracts^2,18,20,23^ to be used as cross-linkers in gelatin modification.

To reduce pollution problems and stimulate the economy, the production of value-added materials from agro-industrial wastes has become increasingly widespread in recent years.^24^ In this study, the modification of bovine gelatin was carried out with agro-industrial food production wastes such as pomegranate peel, grape pomace and seed, and black and green tea waste, which are suitable for use in foods, are nontoxic, inexpensive, and easily available, and have high polyphenol content. The effect of modification on the textural, rheological, emulsifying, foaming, swelling, and color properties, as well as the total phenolic content and antioxidant activity of gelatin, was discussed.

## 2 Materials and Methods

### 2.1 Materials

Gelatin (bovine skin, 215 bloom) samples were provided from a gelatin production company (Halavet, İstanbul, Türkiye). The pomegranates at the same level of ripeness were sourced from a local producer in the Mersin province of Türkiye. The green tea waste was provided as production waste from a local tea production company (Efor Tea, Trabzon, Türkiye). Black tea and grape pomace and seed wastes were provided by a food and beverage production company (Döhler, Riedstrasse, Germany). All other chemicals and reagents were at least of analytical grade.

### 2.2 Preparation of the Agro-Industrial Food Wastes (AFW)

The preparation of AFW was carried out according to the method of Songkhai and Sompongse^25^ with some modifications. The agro-industrial wastes were washed with water to remove all unwanted materials (dust, soil, etc.) and dried in an oven at 50 °C for 24 h. Dried waste samples were ground to facilitate extraction using a colloid mill (LD-600N, Lingdong, China) to a sieve size of 6 mm. Then, the coarse materials were subjected to grinding (King, K-444 Grindex, Istanbul, Türkiye) and passed through a 35 mesh sieve. The obtained waste powders were vacuum-sealed and stored at room temperature (25 °C) in a desiccator until further analysis and preparation of the ethanol extracts.

### 2.3 Phenolic Extraction of AFW

Waste phenolic extracts were prepared according to the method of Hoque et al.^26^ with some modifications. The AFW powders (50 g) were mixed with 500 mL of 70% (v/v) ethanol using a homogenizer (VELP Scientifica, OV-5, Italy) at 10,000 rpm for 2 min and an ultrasonic water bath (Daihan, WUC-D10H, South Korea) at 300 W for 2 min, respectively. The homogenized mixtures were then stirred continuously using a magnetic stirrer at 500 rpm and 40 °C for 1 h and subsequently centrifuged at 9,000*g* for 15 min at 25 °C using a refrigerated centrifuge (Hettich, Universal 320R, Germany) to separate undissolved matter. The residues were collected, and an additional extraction was carried out using an equivalent volume of solvent. The resulting supernatants were then combined and filtered through Whatman filter paper No.4 (Whatman International Ltd., Maidstone, U.K.). Then, the combined supernatants were evaporated under vacuum using a rotary evaporator (Buchi, R-100, Switzerland) at 40 °C to remove the ethanol.

To obtain ethanolic extract powders, the supernatants were lyophilized using a freeze-dryer (Teknosem, TRS-2, Türkiye) at −55 °C for 4 days. The agro-industrial waste extract powders, termed pomegranate peel extract (PPE), grape pomace and seed extract (GPE), black tea extract (BTE), and green tea extract (GTE) were vacuum-packed (Sonkaya, SMVK150G, Türkiye) and stored at room temperature (25 °C) in a desiccator until further analysis. The yields of PPE, GPE, BTE, and GTE were 40.74, 14.41, 6.71, and 4.22 g 100 g^–1^ waste powder (on a dry basis), respectively.

### 2.4 Oxidization of the AFW Extracts (AFWEs)

The oxidization process was carried out according to the method of Strauss and Gibson^11^ with some modifications. The AFWEs (0.4 g) were dissolved in 16 mL of distilled water at 40 °C, then centrifuged at 7,000*g* for 10 min at 25 °C using a refrigerated centrifuge (Hettich, Universal 320R, Germany) to remove nonwater-soluble fractions. Then, the pH of the supernatant was adjusted to 9 using 1 mol/L NaOH.

To oxidize the phenolic compounds in the remaining extracts and convert them into quinones, the solutions were stirred at 40 °C and 300 rpm for 1 h using a magnetic stirrer, while simultaneously bubbling the solutions with oxygen with a purity of ≥99.9%. During oxygenation, the pH of the solutions was measured using a pH meter (Ohaus ST3100, NJ) and kept constant at 9 by dropwise addition of 1 mol/L NaOH. The temperature of the solutions was also monitored using an alcohol thermometer and maintained constant at 40 °C by adjusting the magnetic stirrer temperature if necessary. After the oxidation, the volume of the oxidized AFWE solution was adjusted by using distilled water to obtain a concentration of 2% (w/v) AFWE.

### 2.5 Modification of the Gelatin Gels by Covalent Interaction

Bovine gelatin (215 bloom) samples were chemically modified via covalent interaction with oxidized AFWEs (PPE, GPE, BTE, and GTE) at different concentrations (1, 3, and 5% w/w, based on gelatin). To modify the gelatin samples with AFWEs, the method of Strauss and Gibson^11^ was employed with some modifications. After being hydrated at room temperature for 1–4 h, the bovine gelatin (3.335 g) was dissolved in distilled water (40 mL) using a water bath at 60 °C for 15 min. The pH of the gelatin solution was adjusted to 9 with 1 mol/L NaOH, and then the solution was stirred for another 15 min. The gelatin solutions were cooled to 40 °C, and then the prepared oxidized AFWE solutions were slowly added to obtain different final concentrations of 1, 3, and 5% (w/w, based on gelatin). The oxidization process was performed as previously described, and then the modified gelatin solutions were neutralized (pH 7) using 1 mol/L HCl. The volume of the final solution was adjusted by using distilled water to obtain a concentration of 6.67% (w/v) gelatin.

The resulting mixture was allowed to cool for about 15 min at room temperature and then cooled at 10 °C and 50% relative humidity for 17 h to form hydrogels. The unmodified gelatin (control) samples were prepared in the same manner but without AFWE. The control and modified gelatin gels were subjected to various analyses.

### 2.6 Textural Analysis (Bloom Strength)

After the conditioning, the bloom strength analysis of the control and modified gels was performed according to the Gelatin Manufactures Institute of America (GMIA) standards^27^ with slight modification. The texture analyzer (TA.HDplus Texture Analyzer, Stable Micro System, Surrey, U.K.) equipped with a 5 kg load cell and an AOAC plunger (P/0.5) was used. The pretest, test, and post-test plunger speed was 0.5 mm/s. The trigger force was 5 g. The force (in grams) required to penetrate the AOAC plunger into the gel sample at 4 mm was recorded as the bloom strength. Analyses were conducted at five distinct locations.

### 2.7 Dynamic Rheological Measurements

Rheological measurements were conducted using a stress- and strain-controlled rheometer (Anton-Paar MCR-302, Graz, Austria) equipped with a Peltier system and a parallel plate (PP25, 25 mm diameter). The gap setting and sample volume were 1 mm and 500 μL, respectively. The periphery of the sample was covered with a thin layer of silicon oil to prevent evaporation during the measurements. All analyses were carried out in duplicate.

#### 2.7.1 Gelling and Melting Temperatures

The temperature sweep analysis was performed according to the method of Kuan et al.^28^ with some modifications. Before the analysis, gel samples were heated at 55 °C for 15 min using a water bath. The strain and frequency values were 1% and 1 Hz, respectively, and were within the linear viscoelastic region. Initially, the preheated samples were conditioned at 50 °C for 10 min to erase thermal history.^29^ Then, the samples were cooled from 50 to 5 °C and held at 5 °C for 5 min before being heated again to 50 °C, both at a scanning rate of 0.5 °C/min. The crossover points of storage modulus (*G*′) and loss modulus (*G*″) during cooling and heating scans were defined as the gelling and melting temperatures, respectively.

#### 2.7.2 Gelation Kinetics

After the temperature sweep analysis, a time sweep analysis was conducted by using the method of Fonkwe et al.^30^ with some modifications. The samples were cooled from 50 to 5 °C at a scanning rate of 0.5 °C/min and then maintained at 5 °C for 180 min at a strain of 1% and a frequency of 1 Hz. During the analysis, the *G*′ and *G*″ values were recorded. To determine the gelation rate, the *G*′ values over time were fitted to a logarithmic equation
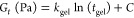
1where *G_t_* is the value of *G*′ at time *t*, *k*_gel_ is the gelation rate constant, *t*_gel_ is gelation time, and *C* is a constant. The time required (*t*_model_) to reach the storage modulus of the control gelatin at the end of time sweep analysis (*G*′_ref_) was calculated using the following equation:
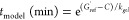
2

#### 2.7.3 Determination of Gel Strength

The frequency sweep analysis was performed following the time sweep analysis. The *G*′, *G*″, and storage compliance (*J’*) values were recorded over a frequency range of 0.1–10 Hz at 5 °C. The gel strength value was calculated using the following equation:
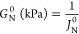
3where *G*_N_^0^ is the gel strength of the sample and *J*_N_^0^ is the storage compliance at the frequency with minimum *G*″.^31^

### 2.8 Fourier Transform Infrared (FTIR) Spectroscopy

FTIR spectra of gel samples were determined by using a spectrometer (Bruker, ALPHA II, Germany) equipped with an attenuated total reflection (ATR) accessory. The spectra were collected in a wavenumber range of 400–4,000 cm^–1^ at a resolution of 4 cm^–1^ with 16 scans per minute.^32^

### 2.9 X-ray Diffraction Studies (XRD)

The XRD analysis was performed according to the method of Zhao et al.^3^ with slight modifications. XRD patterns of freeze-dried samples were obtained using an X-ray diffractometer (Bruker D8 Discover, Karlsruhe, Germany) equipped with a Ni-filtered Cu Kα radiation source (λ = 1.5418 Å) and operating at irradiation conditions 40 kV and 40 mA. Data were recorded in the angular range of 2θ = 5–40°, at a scanning rate of 2°/min, and a step size of 0.02°.

### 2.10 Scanning Electron Microscopy (SEM)

The surface morphology of the gel samples was characterized using SEM (Carl Zeiss, GeminiSEM 300, Germany).

### 2.11 Determination of Degree of Cross-Linking

The degree of cross-linking was evaluated according to the procedure described by Sheu et al.^33^ with some modifications. To a 125 μL of 40 mg/mL gel sample, 1 mL of 4% (w/v) sodium bicarbonate solution and 1 mL of 0.1%^34^ (w/v) freshly prepared TNBS (2,4,6-trinitrobenzenesulfonic acid) solution was added. The mixture was incubated at 40 °C for 3 h in a water bath. After incubation, 3 mL of 6 mol/L HCl was added to terminate the reaction, and the temperature was adjusted to 60 °C for 90 min. Then, the mixture was diluted at a ratio of 1–20 with distilled water^35^ and cooled at room temperature for 15 min. The absorbance of the diluted solution was measured at 346 nm using a ultraviolet–visible (UV–vis) spectrophotometer (Rigol Ultra-3660, Beijing, China). The cross-linking degree was calculated as follows:^34^
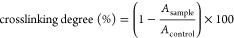
4where *A*_sample_ is the absorbance of the cross-linked gels and *A*_control_ is the absorbance of the control gel.

### 2.12 Determination of *L*, *a*, *b*, and Δ*E* Values

Gel samples were heated at 55 °C for 15 min using a water bath and transferred to a 20 mL optical cuvette. The darkness/lightness (L*), greenness/redness (a*), blueness/yellowness (b*), and total color difference (ΔE*) of gel samples were measured using a spectrophotometer (Ultrascan Vis, HunterLab). D65 illuminant was used as the light source. The spectrophotometer was standardized using a white plate (L* 94.35, a* – 0.05, and b* 0.41).

### 2.13 UV Spectroscopy, Turbidity, Color, and Clarity

UV spectra of gel samples were recorded in a wavelength range of 300–800 nm using a UV–vis spectrophotometer (Rigol Ultra-3660, Beijing, China). Turbidity,^19^ color, and clarity^27^ values were measured by reading the percentage transmittance at 360, 450, and 620 nm, respectively.

### 2.14 Total Phenolic Content (TPC)

The TPC of AFWEs and gel samples were measured according to the Folin-Ciocalteu method with some modifications.^36^ In brief, 0.3 mL of an appropriately diluted sample solution was mixed with 1.5 mL of Folin-Ciocalteu reagent (10% v/v) using a vortex mixer. The mixture was allowed to stand at room temperature for 5 min. Then, 1.2 mL of sodium carbonate (7.5% w/v) was added and mixed. After incubation at 40 °C for 120 min, the absorbance was measured at 760 nm using a UV–vis spectrophotometer (Shimadzu UV-1800, Japan). Gallic acid solutions in a range of 10–100 μg/mL were used to obtain a standard curve (*R*^2^ = 0.9981). The blank was prepared in the same manner, except distilled water was used instead of the extract. The TPC of the samples was expressed as milligrams of gallic acid equivalents per gram of dry weight of the samples (mg GAE/g dw).

The TPC analysis was used to determine the conversion of phenolic compounds in the AFWEs to quinones. The differences between the TPC of AFWEs before and after oxidation were given as percentages and expressed as conversion ratios.^26^

### 2.15 Antioxidant Activity by ABTS and DPPH Methods

The ABTS (2,2′-azino-bis(3-ethylbenzothiazoline-6-sulfonic acid)) radical scavenging activity of gel samples was determined according to the method of Re et al.^37^ with some modifications. In brief, 0.2 mL of an appropriately diluted sample solution was mixed thoroughly with 2.8 mL of an ABTS+ solution using a vortex mixer. The solution was then incubated at 40 °C for 6 min in the dark. The absorbance was measured at 734 nm by using a UV–vis spectrophotometer (Rigol Ultra-3660, Beijing, China). The standard curve of Trolox (30 and 300 μM) was prepared in the same manner. The result was expressed as mmol of Trolox equivalent per kg of dry gel sample (mmol of TE/kg of dw).

The DPPH (2,2-diphenyl-1-picrylhydrazyl) radical scavenging activity of gel samples was measured according to the method proposed by Brand-Williams et al.^38^ with some modifications. Gel samples (0.4 g) were dissolved in 10 mL of deionized water, and 0.3 mL of each sample was mixed with 2.7 mL of DPPH solution in ethanol (0.04 mg/mL). The mixture was incubated at 40 °C for 30 min in the dark. The absorbance was recorded at 517 nm by using a UV–vis spectrophotometer (Rigol Ultra-3660, Beijing, China). The DPPH radical scavenging activity (RSA) was calculated as follows:^39^
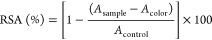
5where *A*_sample_ is the absorbance of the sample, *A*_color_ is the absorbance of the color control, and *A*_control_ is the absorbance of the control.

### 2.16 Determination of Swelling Ratio

About 2 g of gel samples were placed into a 100 mL plastic sample cup and dried to constant weight (*W*_1_) in a vacuum oven at 40 °C. To the dried sample, 20 mL of 0.05 M phosphate buffer (pH 7) was added and allowed to equilibrate for 4 h. After the buffer solution was decanted, the swollen gel samples were blotted with filter paper and weighed (*W*_2_). The swelling ratio was calculated using the following equation:^11^
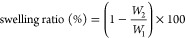
6

### 2.17 Determination of Emulsifying Properties

The emulsion activity index (EAI) and emulsion stability index (ESI) of gel samples were determined according to the method of Aewsiri et al.^21^ with some modifications. To 2 mL of sunflower oil, 6 mL of gel solution (1% (w/v) protein) was added, and the mixture was homogenized using a homogenizer (VELP Scientifica, OV-5, Italy) at 10,000 rpm for 2 min. After homogenization, 25 μL of emulsion was diluted with 4,975 μL of a 0.1% (w/v) sodium dodecyl sulfate (SDS) solution at 0 and 10 min. Then, the mixture was mixed using a vortex mixer, and absorbance was measured at 500 nm using a UV–vis spectrophotometer (Rigol Ultra-3660, Beijing, China). EAI at 0 and 10 min was calculated as follows:
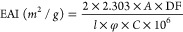
7where *A* is the absorbance at 500 nm, *l* is the path length of the cuvette (*m*), DF is the dilution factor, φ is the oil volume fraction, and *C* is the gelatin concentration (g/m^3^). ESI was calculated using the following equation:^40^
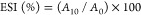
8where *A*_0_ and *A*_10_ are the absorbances at 500 nm at 0 and 10 min, respectively.

### 2.18 Determination of Foaming Properties

The foaming capacity (FC) and foaming stability (FS) of gel samples were determined according to the method of Ghorani et al.^41^ with some modifications. 50 mL of gel solution (0.2% (w/v) protein) was transferred into a 100 mL graduated cylinder and mixed using a homogenizer (VELP Scientifica, OV-5, Italy) at 14,000 rpm for 1 min at room temperature. FC and FS were calculated using foam volumes at 0 and 15 min after mixing, respectively
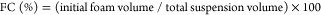
9
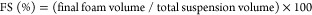
10

### 2.19 Statistical Analysis

The data were expressed as the mean ± standard deviation (SD). Statistical analyses were carried out using R software (version 4.3.2; R Foundation for Statistical Computing, Vienna, Austria). Two-way analysis of variance (ANOVA) was used to analyze the data, and differences between means were evaluated with the Duncan test at a 0.05 significance level. The Pearson correlation test was used to correlate all of the dependent variables. Correlation coefficient values are given in Figure S3.

## 3 Results and Discussion

### 3.1 Bloom Strength

**Figure 1:**
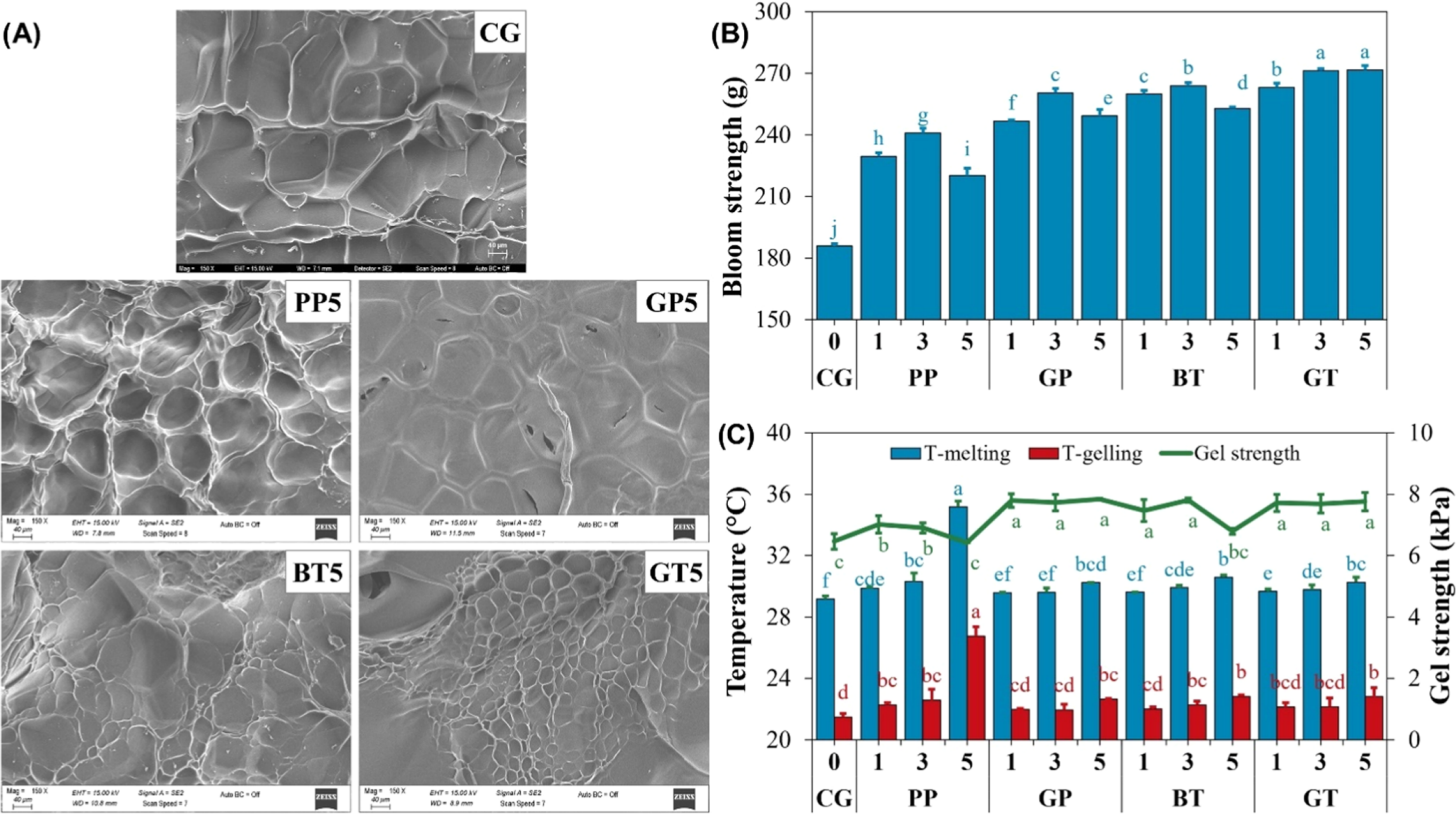
Surface morphology at 150× magnification (A), textural (B), and rheological analysis (C) results of the control and modified gels. CG: control gelatin (without extract); PP: gelatin cross-linked with pomegranate peel waste extract; GP: gelatin cross-linked with grape pomace and seed waste extract; BT: gelatin cross-linked with black tea waste extract; GT: gelatin cross-linked with green tea waste extract; 1–3–5; relevant extract ratio (%) used in the modification.

Bloom strength is one of the key parameters used in determining the usage area and commercial value of gelatin.^12,42^ The results showed that the type of AFWE and concentration had a pronounced effect on the bloom strength of the gelatin gels (Figure 1B). Modifying gelatin with AFWEs significantly increased the bloom strength compared to the control sample (186 g) (*p* < 0.05). Among the modified gels, PP5 had the lowest bloom strength with a value of 220 g, while GT5 had the highest with a value of 272 g. The bloom strength of modified gels increased up to 3% AFWE concentration and decreased, except for GT, over this concentration. These findings were in agreement with the previous studies^1,3,10−12,19,43^ on the bloom strength of gelatin cross-linked with pure phenolic compounds and plant/waste phenolic extracts.

Quinones, resulting from the transformation of phenolic compounds in the presence of oxygen under alkaline conditions, form covalent bonds with the amino and sulfhydryl side chains of polypeptides.^10,11^ In the first stage, the number of covalent bonds increases, and a stronger gel network is formed, resulting in higher gel strength. After reaching the critical point, gel strength begins to decrease as the polyphenol ratio increases due to the precipitation of gelatin and unreacted extract content.^3^ In another study, Kosaraju et al.^22^ stated that the complete denaturation of the triple helix structure of gelatin as a result of cross-linking reduces the gel strength.

Temdee et al.^44^ attributed the higher polyphenol-protein interaction to phenolic compounds with smaller sizes and a greater number of hydroxyl groups. Zhao et al.^3^ stated that the concentration, chain size, and molecular weight distribution of gelatin affect the gel strength. It is thought that the difference in the gel strength in our study is related to the total phenolic content and phenolic composition of the AFWEs.

In our preliminary study, there were no significant changes in the textural properties of the modified gels without oxygen bubbling (data not shown). Temdee and Benjakul^45^ also reported the same result in their study. It has been concluded that without the presence of oxygen, cross-linking occurs to a limited extent and is insufficient to change the properties of gelatin.

### 3.2 Dynamic Rheological Properties

#### 3.2.1 Gelling and Melting Temperatures

**Figure 2:**
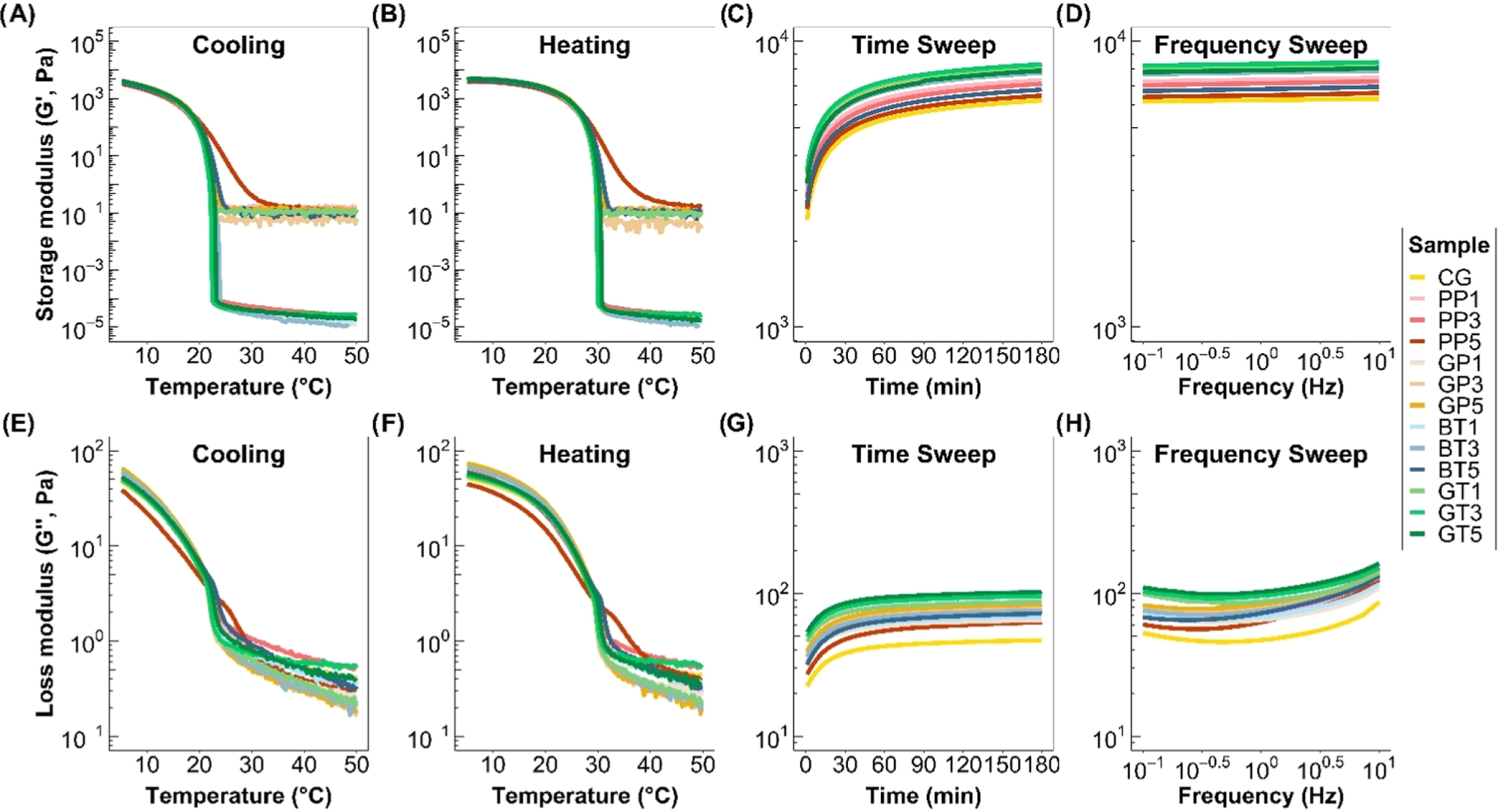
Rheological behavior of the control and modified gel samples. (A–D): storage modules (*G*′, Pa) values for each analysis; (E–H): loss modulus (*G*′’, Pa) values for each analysis; CG: control gelatin (without extract); PP: gelatin cross-linked with pomegranate peel waste extract; GP: gelatin cross-linked with grape pomace and seed waste extract; BT: gelatin cross-linked with black tea waste extract; GT: gelatin cross-linked with green tea waste extract; 1–3–5; relevant extract ratio (%) used in the modification.

The transformation from a liquid to a solid state in a cross-linkable material is termed the sol–gel transition. Gelatin gels are formed through physical interactions that occur along the chain, rather than at individual points.^22^ Rheological behavior of control and modified gel samples is shown in Figure 2. The crossover points of storage modulus (*G*′) and loss modulus (*G*″) during cooling (Figure 2A,E) and heating (Figure 2B,F) scans were defined as the gelling and melting temperatures, respectively. Compared to the control sample, by far the highest gelling and melting temperature increase was observed in the PP5, with 5.29 and 6.01 °C, respectively (Figure 1C). At 5% AFWE concentrations, gelling and melting temperatures increased significantly in all modified gels (*p* < 0.05). Only the PP showed an increase in both gelling and melting temperatures at all extract concentrations. A very strong correlation (*r* = 0.99) was found between the gelling and melting temperatures (Figure S3). In addition, there was a strong correlation between melting and gelling temperatures and the TPC, ABTS, DPPH, and EAI (0.76–0.94).

Lu et al.^46^ stated that the increase in electrostatic interactions reduces the distance fluctuation between particles and improves steric effects. They stated that this situation led to the development of three-dimensional (3D) solid network structures and changes in the thermal and rheological properties of the gels. According to Kaewdang and Benjakul,^1^ the increase in chemical junctions is responsible for the rise in gelling and melting temperatures. The described interactions and network structures in the literature were confirmed by SEM (Figure 1A) and microscope images (Figure S1). Bedis Kaynarca et al.^18^ carried out mixture design modeling, mixing fish skin gelatin with a blend of three different agricultural waste extracts (grape pomace, pomegranate peel, and green tea) at a total ratio of 20% (on a dry gelatin basis). They conducted the modification without introducing oxygen and without adjusting the pH to an alkaline level, resulting in an increase of 2.22–9.41 °C in the melting temperature and 0.98–3.05 °C in the gelling temperature. In our PP5 gel sample, although 4 times lower PPE was used in the modification, a higher gelling temperature increase and a similar melting temperature increase were obtained. Observations indicate that the direct utilization of phenolics results in a lesser increase in melting and gelling temperatures of gelatin compared with their use after conversion into quinones using the alkaline method.

While the *G*′ and *G*″ values of the PP5 sample showed a more gradual change during the cooling and heating stages, the other gels showed a sharper increase or decrease (Figure 2A,B,E,F). Zhu et al.^47^ stated that clustering affects the rheological properties of proteins. It is believed that the high quinone content causes aggregation in the gelatin, leading to a more heterogeneous gel structure. The gradual change in the PP5 sample occurred as a result of the heterogeneous gel structure formed after modification. Similar behavior was reported by Kosaraju et al.^22^ in gels cross-linked with caffeic acid. They stated that chemical cross-linking increases the resistance of gelatin gels to thermal degradation and the breaking of physical interaction occurred more gradually over a wider temperature range compared to control gelatin gels.

### 3.2.2 Gelation Kinetics

Time sweep analyses were conducted to evaluate the gelation kinetics of gelatin (Figure 2C,G). The *G*′ values and therefore the gel strength of the control and modified gel samples increased with increasing gelation time. Similar results were reported in other studies.^15,18,22,43^

The *G*′ data fitted with the logarithmic (eq 1) and model parameters are given in Table 1. All equations had statistically significant (*p* < 0.001) model parameters (*k*_gel_ and *C*) and relatively high adjusted *R*^2^ (>0.99). With the covalent modification of gelatin, the gelation rate constant (*k*_gel_) increased in all types of AFWEs and concentrations compared to that of CG. The gelation rate increased by more than 20% for all concentrations of GP and GT together with BT1 and BT3. For PP and BT samples, the gelation rate decreased with increasing extract concentration. In modified gel samples, *t*_model_ values dramatically decreased compared to those of the control sample. The highest decrease in the *t*_model_ value occurred in the GT3 sample with 85.70%, while the lowest decrease with 23.19% was seen in the PP5 gel. These results showed that exceeding the critical level in cross-linking negatively affected the *k*_gel_ and *t*_model_ as well as the gel strength. Similar to the results in our study, Bedis Kaynarca et al.^18^ reported that modification of gelatin using agricultural waste extracts increased the *k*_gel_ and decreased the *t*_model_. The increase in gelation rate over time can be attributed to the presence of physical cross-links, specifically ionic and hydrogen bonds.^43^

**Table 1:** Parameters ± Standard Errors of the Fits of the Logarithmic Equation together with Adjusted Determination Coefficient, Root-Mean-Square Error, and *t*_model_ Values^a^

sample	extract ratio (%)	adjusted *R*^2^	RMSE (Pa)	*k*_gel_^b^ (Pa/min)	*C*^b^ (Pa)	*t*_model_ (min)
CG	0	0.9922	73.7	874.9 ± 5.8	1712.4 ± 25.0	180.0
PP	1	0.9923	83.7	998.1 ± 6.6	2187.8 ± 28.4	58.9
	3	0.9921	82.7	975.6 ± 6.5	2068.9 ± 28.1	73.1
	5	0.9924	73.4	881.9 ± 5.8	1909.1 ± 24.9	138.3
GP	1	0.9923	91.0	1083.0 ± 7.1	2463.7 ± 30.9	33.2
	3	0.9923	91.1	1084.4 ± 7.2	2439.7 ± 30.9	33.8
	5	0.9922	89.3	1060.6 ± 7.0	2399.1 ± 30.3	38.0
BT	1	0.9924	88.4	1062.0 ± 6.9	2356.4 ± 30.0	39.3
	3	0.9922	88.8	1051.1 ± 7.0	2349.6 ± 30.1	41.1
	5	0.9919	80.7	940.4 ± 6.3	1931.8 ± 27.4	99.3
GT	1	0.9924	88.7	1066.3 ± 7.0	2675.3 ± 30.1	28.7
	3	0.9925	89.9	1086.1 ± 7.1	2727.8 ± 30.5	25.7
	5	0.9921	91.8	1080.1 ± 7.2	2333.6 ± 31.1	37.8

a Data were expressed as mean ± standard error. CG: control gelatin (without extract); PP: gelatin cross-linked with pomegranate peel waste extract; GP: gelatin cross-linked with grape pomace and seed waste extract; BT: gelatin cross-linked with black tea waste extract; GT: gelatin cross-linked with green tea waste extract; adjusted *R*^2^: adjusted determination coefficient, RMSE: root-mean-square error; *k*_gel_: gelation rate constant in Pa/min; C: model intercept in Pa; *t*_model_: time (min) required to reach the storage modulus value of the control gelatin sample at 180 min for each model.

b *p* < 0.001 for all coefficients.

### 3.2.3 Gel Strength

The mechanical strength, deformation, and stability of gelatin gels were evaluated using a frequency sweep test.^18^ Following the time sweep analysis, the frequency sweep test (Figure 2D,H) was performed, and the gel strength values (in kPa) were calculated using eq 3 (Figure 1C). All gel samples displayed shear thinning behavior, in which the frequency increased and complex viscosity (η*) decreased linearly (Figure S2).^16^ The complex viscosity (η*) of the control gel increased with the addition of AFWE, indicating that the overall resistance to flow increased.^48^ Throughout the frequency test, the loss factor (*G*′′/*G*′) for all samples was <0.04, indicating a stable and solid-like gel structure. As the frequency increased, the *G*′ values of all samples exhibited a linear increase, while the *G*′′ values initially showed a slight decrease followed by an exponential rise. The *G*′ values at the moment when the *G*′′ value was at its minimum were recorded as the gel strength of the samples. Increasing the PP and BT extract ratio to 5% decreased the gel strength due to the heterogeneous structure formed as a result of gelatin agglomeration. Sow et al.^15^ reported that gel strength is related to cross-linking density, and gel strength decreases with increasing size of complex coacervates. Except for PP5 and BT5, gel strength values increased compared to CG. In other studies where gelatin was modified with caffeic acid^43^ or phenolic extracts,^18,19^ higher gel strength was reported compared to control samples. A high correlation was detected between bloom strength (Figure 1B) and gel strength (Figure 1C) values (*r* = 0.75) (Figure S3).

### 3.3 FTIR Spectroscopy

**Figure 3:**
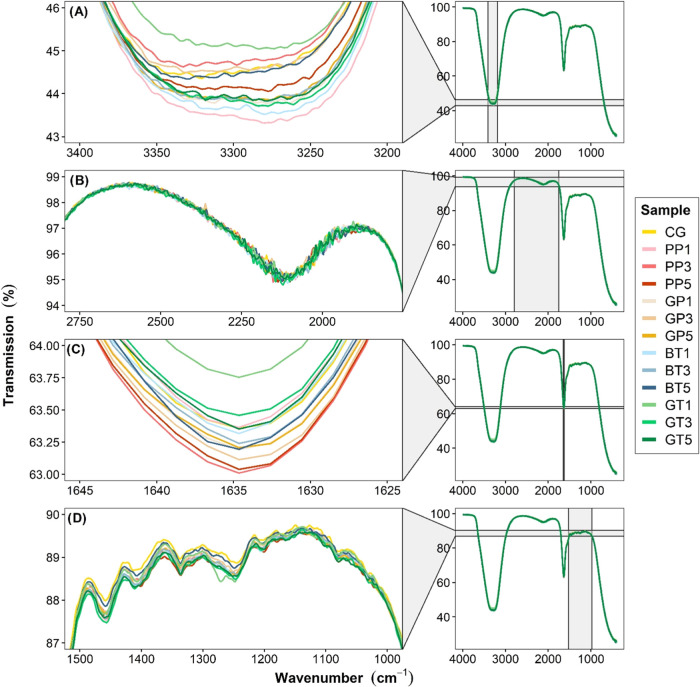
FTIR spectra of the control and modified gel samples. (A–D): zoomed-in images of areas in various regions in the FTIR spectra; CG: control gelatin (without extract); PP: gelatin cross-linked with pomegranate peel waste extract; GP: gelatin cross-linked with grape pomace and seed waste extract; BT: gelatin cross-linked with black tea waste extract; GT: gelatin cross-linked with green tea waste extract; 1–3–5; relevant extract ratio (%) used in the modification.

FTIR spectra provide insights into the molecular-level conformational changes and functional groups of gelatin samples.^3^ FTIR spectra of gel samples are shown in Figure 3A–D. The alterations in the secondary structure of control and modified gelatin were examined using five band regions: 3200–3400 cm^–1^ (amide A representing N–H stretching and −OH bonding), 2000–2250 cm^–1^ (amide B representing –NH_3+_ and = C–H stretching), 1600–1650 cm^–1^ (amide I representing C=O stretching, N–H bending and hydrogen bonding coupled with COO−), 1400–1500 cm^–1^ (amide II representing N–H bending and C–N stretching), and 1200–1300 cm^–1^ (amide III representing N–H bending).^18,23,44,49,50^ While the intensities of amide A in samples GT1 and PP3 were lower than those of the control samples, the other modified gel samples exhibited higher intensities (Figure 3A). No significant change was observed in the amide B region among the gel samples (Figure 3B). In the amide I region, samples PP3 and PP5 had the highest intensity, while GT1 distinctly had the lowest intensity (Figure 3C). Minor changes in the amide II and amide III regions were observed for different gel samples (Figure 3D). Slight changes in the properties of the amide bands of gelatin were detected after modification with different AFWEs. Similar results were also reported by Bedis Kaynarca et al.^18^

### 3.4 X-ray Diffraction

**Figure 4:**
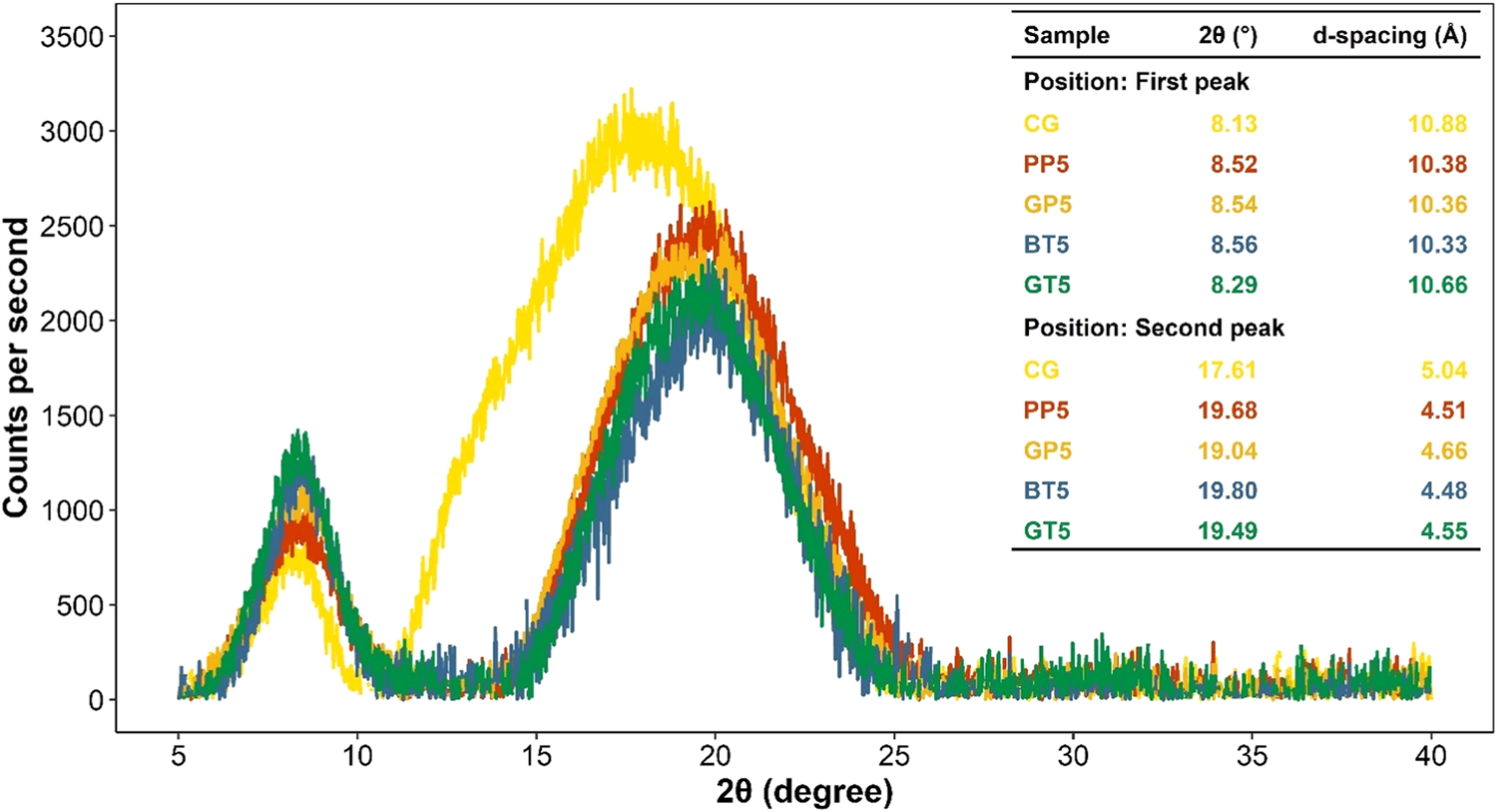
X-ray diffraction (XRD) diagrams of freeze-dried gelatin gels with and without oxidized phenolic extracts. CG: control gelatin (without extract); PP: gelatin cross-linked with pomegranate peel waste extract; GP: gelatin cross-linked with grape pomace and seed waste extract; BT: gelatin cross-linked with black tea waste extract; GT: gelatin cross-linked with green tea waste extract; 5: relevant extract ratio (%) used in the modification.

Figure 4 shows the X-ray diffractograms of the control and modified gelatin samples along with the 2θ° and *d*-spacing (Å) values for each peak. In general, the XRD patterns of gelatin display two peaks. While the first peak represents the distance between the molecular chains, the second peak is associated with diffused scattering.^3,10,51^ Peak positions (2θ°) of control gelatin were 8.13 and 17.61° for the first and second peaks, respectively. When oxidized AFWEs were introduced, both peak positions of gelatin shifted toward higher 2θ° values, which resulted in lower *d*-spacing values. Peak shifting was more dramatic for the second peak. Compared to the control sample, the *d*-spacing value decreased from 10.88 to 10.33 Å for the first peak and 5.04 to 4.48 Å for the second peak. There was no substantial difference between the modified samples. Owing to the increase in intermolecular interactions between amino and carboxyl groups in gelatin and the hydroxyl groups in oxidized phenolic extracts, a denser gel structure with limited molecular movement could be obtained.^3,10,51,52^ This was confirmed by the decrease of the *d*-spacing values in modified samples. The XRD findings were in agreement with the SEM (Figure 1A) and light microscopic images (Figure S1). Our results are in line with the literature.^3,10,51,52^

### 3.5 Microstructure

The surface microstructure of gel samples at 5% AFWE concentrations at 150× magnification is given in Figure 1A. The configuration and bonding patterns of molecules in gel matrices are influential factors in the strength of gelatin gels.^45^ The control gel had thin strands. The PP5 sample had by far the thickest strands and similar pore size as CG. GP5 gel also had a similar pore size compared to CG but had thicker strands. In some studies, modification of gelatin using oxidized phenolic extracts had been reported to increase pore size and strand thickness.^44,45^ There was no significant change in the strand thickness in the BT5 and GT5 gels. The GT5 gel exhibited smaller pore sizes and a uniform network. This structure can be associated with the GT5 sample having the highest gel strength and gelation rate. On the other hand, the increase in strand thickness in the PP5 sample can be linked to the observed increase in melting and gelling temperatures.

### 3.6 Degree of Cross-Linking

Quantification of free amino groups in gelatin samples is achieved through the reaction between the reagent TNBS and primary amino groups.^3,12^ It is suggested that the number of free amino groups in gelatin decreases by interacting with quinones formed during oxygenation under alkaline conditions.^21,44^ The change in free amino content before and after modification was expressed as the degree of cross-linking. Cross-linking degree values, from highest to lowest, were determined as 9.30 ± 1.20, 8.45 ± 1.12, 8.07 ± 1.21, and 6.60 ± 1.13% for GT5, BT5, PP5, and GP5 samples, respectively. In some literature studies, it has been stated that the use of oxidized phenolic compounds or phenolic extracts leads to a reduction in the free amino content of gelatin samples.^3,21,44,45,50^ Oxidized ferulic acid, caffeic acid, and tannic acid were reported to reduce the free amino content of gelatin by 5.99, 21.09, and 9.33%, respectively.^21,50^

Zhao et al.^3^ stated that the degree of cross-linking increased up to a critical phenolic extract concentration and decreased beyond this critical concentration value. They suggested that the agglomeration of gelatin in the presence of high phenolic concentrations affected the results. Although the PP5 sample had the highest phenolic content, it had a lower cross-linking value than the GT5 and BT5 samples, indicating that the critical value for this sample was exceeded.

Aewsiri et al.^21^ stated that the degree of cross-linking depends on the type and concentration of oxidized phenolic compounds used. They also emphasized that the molecular size of phenolics was inversely proportional to the degree of cross-linking.

### 3.7 *L*, *a*, *b*, and Δ*E* Values

L*, a*, and b* along with the total color difference (Δ*E*) values of gels are given in Table 2. Modification of gelatin had a dramatic impact on the color values of gelatin gel. For each type of AFWE, the *L* value decreased and the a* value increased with increasing concentration. The *b* value in modified gels increased significantly compared to the CG. The results showed that with an increasing concentration of AFWE, the gels exhibited an increase in redness and yellowness, while darkness decreased. Similar results were reported in other studies.^1,12,44,45^ Temdee and Benjakul^45^ modified the cuttlefish skins gelatin using oxidized Kiam wood and cashew bark extracts at different concentrations of extracts (1–8%, w/w). They reported a decrease in the L* (54.10–16.50) and b* (36.40–11.10) values and an increase in the a* (−0.60–27.3) value with increasing concentration compared with the control gel.

**Table 2:** *L*, *a*, *b*, and Δ*E* Color Values of Control and Modified Gelatin Gels^a^

sample	extract ratio (%)	L*	a*	b*	Δ*E*
CG	0	94.47 ± 0.16^a^	–1.04 ± 0.02^k^	7.22 ± 0.37^l^	0.00 ± 0.00j
PP	1	76.83 ± 0.13^d^	6.38 ± 0.39^h^	74.72 ± 0.58^g^	70.16 ± 0.91^f^
	3	59.09 ± 0.13^g^	26.10 ± 0.50^d^	93.29 ± 0.31^a^	96.94 ± 0.70^a^
	5	47.29 ± 0.02^j^	34.67 ± 0.01^a^	79.60 ± 0.05^d^	93.13 ± 0.70^b^
GP	1	83.14 ± 0.11^b^	5.07 ± 0.22^i^	37.16 ± 1.75^k^	32.60 ± 1.08^i^
	3	69.54 ± 0.41^e^	21.49 ± 0.26^e^	68.19 ± 1.64^h^	69.62 ± 0.79^f^
	5	59.72 ± 0.45^g^	33.62 ± 0.39^b^	82.01 ± 1.07^c^	89.47 ± 0.20^c^
BT	1	78.36 ± 0.97^c^	7.26 ± 0.82^g^	48.50 ± 2.80^i^	45.09 ± 2.53^g^
	3	59.38 ± 2.11^g^	26.05 ± 1.86^d^	76.39 ± 1.80^f^	82.17 ± 2.52^d^
	5	48.69 ± 2.62^i^	34.05 ± 1.18^ab^	77.91 ± 2.22^e^	91.30 ± 0.29^bc^
GT	1	82.72 ± 0.50^b^	2.57 ± 0.13^j^	44.23 ± 1.59^j^	39.01 ± 1.94^h^
	3	67.95 ± 0.01^f^	16.68 ± 1.21^f^	75.36 ± 0.20^fg^	75.24 ± 0.08^e^
	5	56.35 ± 1.38^h^	27.35 ± 0.40^c^	84.68 ± 0.16^b^	90.89 ± 0.65^c^

a Data were expressed as mean ± standard deviation (*n* = 10). CG: control gelatin (without extract); PP: gelatin cross-linked with pomegranate peel waste extract; GP: gelatin cross-linked with grape pomace and seed waste extract; BT: gelatin cross-linked with black tea waste extract; GT: gelatin cross-linked with green tea waste extract; Δ*E*: total color difference. ^a-l^ Means within the same column with different letters are significantly different at *p* < 0.05.

Quinines formed as a result of the oxidation of polyphenols can undergo condensation reactions, leading to the formation of tannins, which are high molecular weight and brown-colored pigments. The formed tannin pigments can react with the SH and amino groups of proteins, influencing the color characteristics of the gelatin gel.^53^ Total color change (Δ*E*) values increased with an increasing extract concentration for all AFWEs (Table 2). For each level of AFWE concentration, the most significant color change (Δ*E*) was observed in the PP sample.

### 3.8 UV Spectrum, Turbidity, Color, and Clarity

**Figure 5:**
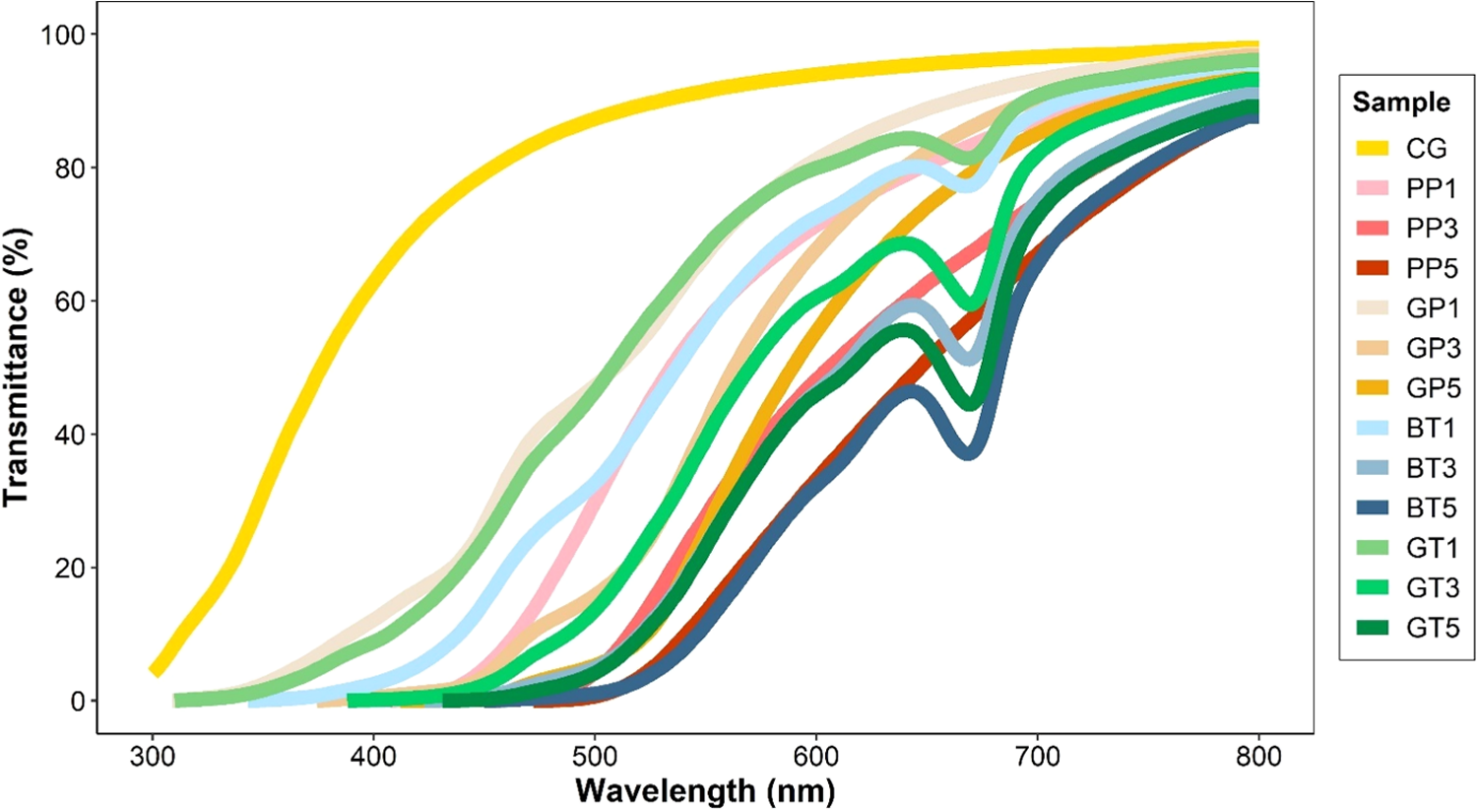
UV spectra of the control and modified gel samples. CG: control gelatin (without extract); PP: gelatin cross-linked with pomegranate peel waste extract; GP: gelatin cross-linked with grape pomace and seed waste extract; BT: gelatin cross-linked with black tea waste extract; GT: gelatin cross-linked with green tea waste extract; 1–3–5; relevant extract ratio (%) used in the modification.

UV spectra of gelatin gels in a wavelength range of 300–800 nm are shown in Figure 5. The transmittance value of the modified gels was lower than the control gel in the entire wavelength range. For each type of AFWE, the transmittance values decreased with increasing concentration. Also, the zero transmittance value shifted toward higher wavelengths. The peak observed at 670 nm is thought to originate from complex structures formed between oxidized catechin derivatives and gelatin. This specific peak can be utilized as confirmation of the use of tea extracts (black and green) in the modified gelatin.

**Figure 6:**
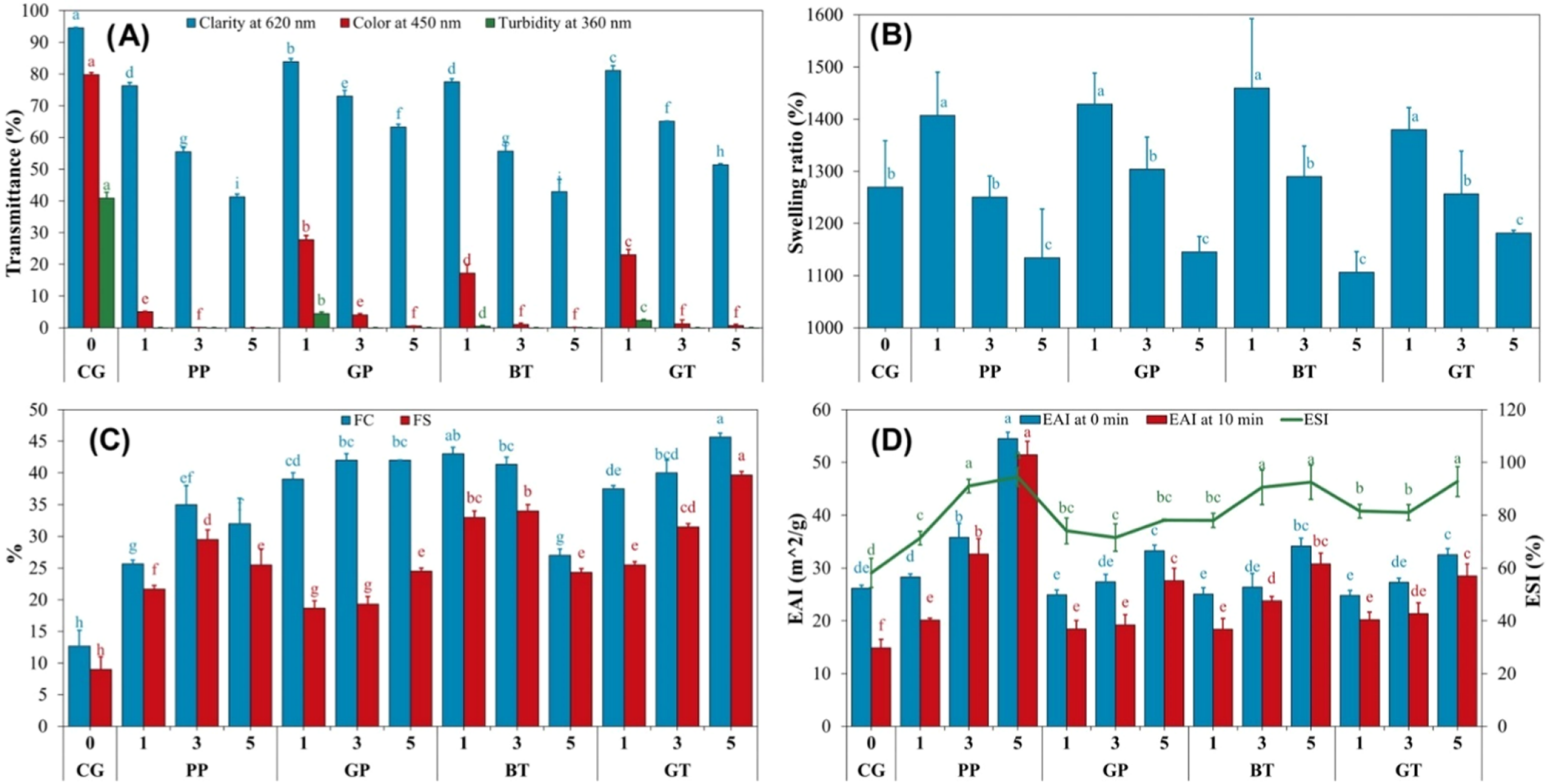
(A) Clarity, color, and turbidity values; (B) swelling ratio; (C) foaming capacity and stability; (D) emulsion activity and stability of the control and modified gel samples. FC: foaming capacity; FS: foaming stability; EAI: emulsion activity index; ESI: emulsion stability index; CG: control gelatin (without extract); PP: gelatin cross-linked with pomegranate peel waste extract; GP: gelatin cross-linked with grape pomace and seed waste extract; BT: gelatin cross-linked with black tea waste extract; GT: gelatin cross-linked with green tea waste extract; 1–3–5; relevant extract ratio (%) used in the modification.

Turbidity, color, and clarity of gel samples measured by reading the percentage transmittance at 360, 450, and 620 nm, respectively, are shown in Figure 6A. These three values decreased dramatically in modified gels compared to the control sample. As the extract concentration increased, color values decreased at all wavelengths. While GP values were closest to the control sample at all concentrations, the PP sample had the furthest values. A correlation of 0.94 was observed between turbidity and color, while a correlation of 0.75 was found between color and clarity. Yasin et al.^19^ reported that the turbidity of chicken feet gelatin gel decreased with the use of basil and lemongrass extract. Color and clarity values are among the quality parameters within the gelatin standards prepared by the Gelatin Manufacturers Institute of America (GMIA).^27^ For commercial bovine gelatin, clarity and color values are required to be greater than 90, and 75%, respectively. While the control gelatin complied with these criteria, all modified gelatins remained below these values. Denaturation of the triple helix structure of gelatin negatively affects the homogeneity of the gel network structure. Turbidity, color, and clarity are closely related to the heterogeneity of the gel network structure.^19^ In our study, a significant negative correlation (*r* = −0.76) was found between bloom strength and turbidity. Strong correlations ((−0.72)-0.98) were also observed between *L*, *a*, *b*, and Δ*E* values and color and clarity (Figure S3).

### 3.9 TPC of AFWEs and Conversion Ratio of Phenolics to Quinones

The TPC of AFWEs and conversion ratio of phenolics to quinones are given in Table 3. TPC of PPE before (572.14 mg GAE/g) and after (394.89 mg GAE/g) oxidation had by far the highest compared to the other AFWEs. In the TPC content of the extract samples before oxidation, the PPE sample is followed by BTE, GPE, and GTE, respectively. The TPC value of AFWEs before oxidation was in agreement with the literature. Derakhshan et al.^54^ found the TPC of three different types of pomegranate peel between 276–413 mg GAE/g extract. In another study, the TPC of PPE was found as 492 mg GAE/g extract.^23^ Bedis Kaynarca et al.^18^ determined the TPC of PP, GP, and GT agricultural waste extracts as 664.56, 194.36, and 1534.32 mg GAE/g, respectively. Bulut et al.^100^ reported the TPC of the GT waste extract between 6.08 and 172.69 mg of GAE/g of dw depending on the ethanol ratio of the solvent. Abdeltaif et al.^55^ found the TPC of the BT waste extract as 152.87 mg GAE/g.

**Table 3:** Total Phenolic Contents of Industrial Waste Extracts before and after Oxidation^a^

extract	TPC-BO (mg GAE/g)	TPC-AO (mg GAE/g)	TPC-converted (mg GAE/g)	conversion ratio (%)
PPE	572.14 ± 2.63^a^	394.89 ± 2.15^a^	177.25 ± 4.77^a^	30.98 ± 0.69^b^
GPE	145.39 ± 1.89^c^	109.70 ± 1.82^b^	35.69 ± 2.77^c^	24.54 ± 1.67^c^
BTE	166.28 ± 0.79^b^	98.94 ± 0.66^c^	67.34 ± 0.98^b^	40.50 ± 0.47^a^
GTE	92.29 ± 0.66^d^	56.92 ± 1.01^d^	35.37 ± 1.66^c^	38.32 ± 1.52^a^

a Data were expressed as mean ± standard deviation (*n* = 6). PPE: pomegranate peel waste extract; GPE: grape pomace and seed waste extract; BTE: black tea waste extract; GTE: green tea waste extract; TPC-BO: total phenolic content of waste extract before oxidation; TPC-AO: total phenolic content of waste extract after oxidation. ^a–d^ Means within the same column with different letters are significantly different at *p* < 0.05.

The number of phenolic compounds converted to quinones in AFWEs was calculated by measuring the differences between TPC before and after oxidation (Table 3). The phenolic equivalent converted to quinones was also given as percentages and expressed as conversion ratio^26^ (Table 3). The total phenolic equivalent converted to quinone was 2.5, 2.5, and 5.0 times higher in the PPE sample compared to those in the GP, GT, and BT samples, respectively. While the quinone conversion ratio was the highest in the BTE and GTE samples with 40.50, and 38.31%, it was the lowest in the GPE sample with 24.54%.

### 3.10 TPC and Antioxidant Activities of Gels

The TPC and antioxidant activity values by ABTS and DPPH are given in Table 4. TPC values were higher in the modified gels (8.41–31.78 mg GAE/g) compared to the CG (7.39 mg GAE/g), consistent with the TPC of AFWEs. The highest TPC was observed in the 5% concentration for each AFWE type, with significant differences between the extract concentrations (*p* < 0.05). Similar trends were also obtained for the ABTS and DPPH radical scavenging activities of control and modified gel samples. The increase in TPC and antioxidant activities was more dramatic for PP, followed by GP, BT, and GT.

**Table 4:** Total Phenolic Content and Antioxidant Activity Values of the Control and Modified Gelatin Gels^a^

sample	extract ratio (%)	TPC (mg GAE/g)	ABTS (μmol TE/g)	DPPH (RSA, %)
CG	0	7.39 ± 0.52^h^	49.03 ± 0.26^g^	1.41 ± 0.45^h^
PP	1	15.33 ± 0.33^d^	82.44 ± 0.51^c^	22.52 ± 0.35^d^
	3	23.25 ± 0.27^b^	141.86 ± 7.87^b^	66.46 ± 1.05^b^
	5	31.78 ± 1.19^a^	214.18 ± 0.00^a^	83.38 ± 3.50^a^
GP	1	9.12 ± 0.25^fg^	62.17 ± 0.68^f^	3.63 ± 0.89^gh^
	3	12.72 ± 0.32^e^	73.79 ± 0.75^d^	15.23 ± 0.90^e^
	5	17.29 ± 0.24^c^	81.04 ± 0.50^c^	27.55 ± 0.98^c^
BT	1	9.53 ± 0.81^f^	61.96 ± 0.88^f^	2.44 ± 0.56^gh^
	3	12.95 ± 0.32^e^	72.04 ± 0.42^d^	8.27 ± 1.49^f^
	5	14.68 ± 0.50^d^	79.98 ± 0.80^c^	9.52 ± 1.09^f^
GT	1	8.41 ± 0.26^g^	51.84 ± 0.30^g^	1.64 ± 0.09^h^
	3	9.85 ± 0.40^f^	60.15 ± 0.27^f^	3.17 ± 1.16^gh^
	5	13.58 ± 0.87^e^	66.96 ± 1.30^e^	4.76 ± 0.24^g^

a Data were expressed as mean ± standard deviation (*n* = 6). CG: control gelatin (without extract); PP: gelatin cross-linked with pomegranate peel waste extract; GP: gelatin cross-linked with grape pomace and seed waste extract; BT: gelatin cross-linked with black tea waste extract; GT: gelatin cross-linked with green tea waste extract. ^a–h^ Means within the same column with different letters are significantly different at *p* < 0.05.

Aewsiri et al.^21^ reported that TPC along with DPPH and ABTS radical scavenging activity values increased with increasing oxidized ferulic acid, caffeic acid, and tannic acid concentration used in the gelatin modification. They also stated that the type and concentration of the oxidized phenolic compound affected the TPC and antioxidant activity values of gelatin at different rates.

The strong antioxidant properties of phenolic compounds are due to their ability to donate hydrogen.^23^ Aewsiri et al.^50^ stated that hydroxyl groups in phenolics play an important role in donating hydrogen and electrons or scavenging radicals and thus terminating the radical chain reaction. The researchers pointed out that the oxidation process did not convert all of the hydroxyl groups in the phenolics to quinones. In a study in which gelatin was modified with oxidized and nonoxidized tannic acid, it was reported that gelatin modified with nonoxidized tannic acid exhibited lower DPPH radical scavenging activity.^56^ These findings showed that the remaining hydroxyl groups after modification of gelatin with oxidized phenolics contribute more to the antioxidative activity.^50^

### 3.11 Swelling, Foaming, and Emulsion Properties of Gel Samples

Swelling, foaming, and emulsion properties are other important parameters for the practical use of gelatins. The swelling ratios of dried control and modified gelatin gels are shown in Figure 6B. It was found that the type of AFWE did not affect the swelling ability of gelatin gels, while the extract concentration played a significant role. Compared to the control gel, the swelling ratio increased at 1% AFWE, remained constant at 3% AFWE, and decreased at 5% AFWE. Strauss and Gibson^11^ reported that the swelling ratio decreased with increasing polyphenol-amino ratio in cross-linked gelatin gels using caffeic acid, grape juice, and coffee. In some studies,^3,19^ it was reported that with increasing extract concentration, the swelling ratio of modified gels decreased and reached a minimum level, and after reaching this minimum level, the swelling ratio increased slightly as the extract concentration increased further.

Foam capacity (FC) and stability (FS) values in all samples increased compared to the control sample (*p* < 0.05) (Figure 6C). Both the highest FC and the highest FS were observed in the GT5 sample. Among the modified gel samples, GP had the lowest FS values. Rahayu et al.^2^ reported an increase in the foaming power of cross-linked gelatin with increasing green tea extract concentration. They also associated a high foaming power with a strong gel network structure. Lu et al.^57^ emphasized that the hydrophobic groups of proteins play an important role in foaming stability. In our study, it is thought that the number of polar groups decreased and the hydrophobicity increased as a result of the interaction of polar groups of gelatin and oxidized phenolic extracts with each other, which increased foaming activity and stability.

Gravitational separation in emulsions occurs due to the growth of oil droplets by coalescence or Ostwald ripening.^58^ Emulsion activity index (EAI) and emulsion stability index (ESI) values increased with increasing AFWE concentration in modified gels (*p* < 0.05) (Figure 6D). The highest EAI was observed in the PP5 sample. Ren et al.^59^ stated that the molecular structure and rheological properties of proteins affect their emulsifying properties. In our study, the PP5 sample had by far the highest melting and gelling temperatures and the thickest strands. It is believed that the improvement in the network structure increases the emulsion stability by preventing coalescence of the oil droplets. Rahayu et al.^2^ reported an increase in the emulsion activity and stability of cross-linked gelatin with the addition of green tea extract. Aewsiri et al.^50^ reported that the emulsion property of gelatin increased with the use of oxidized linoleic acid and decreased with the use of oxidized tannic acid. They emphasized a linear relationship between the surface hydrophobicity of gelatin and its emulsion activity. In another study, Li et al.^60^ stated that the emulsifying capacity of gelatin was improved by increasing the surface hydrophobicity of gelatin with the ultrasound treatment. In our study, it is thought that by cross-linking gelatin with oxidized phenolic extracts, the number of polar groups decreased; thus, the surface hydrophobicity increased, which enhanced the emulsion activity.

## 4 Conclusions

This study aimed to enhance the technological and functional properties of bovine gelatin gels by using different types and concentrations of oxidized agro-industrial food waste extracts. Additionally, the reusability of these agro-industrial food wastes in potential value-added applications such as the modification of gelatin was investigated. The results showed that modification with oxidized waste extracts could increase the gel strength, thermal stability, and gelation rate of gelatin. Compared to the control sample, 46.24% higher bloom strength in the GT5 sample, 5.29 and 6.01 °C higher gelling and melting temperatures in the PP5 sample, respectively, and 85.70% lower *t*_model_ value in the GT3 sample were observed. Additionally, significant improvements were achieved in the total phenolic content, antioxidant activity, foam, and emulsion properties of the modified gels. This study revealed that the oxidized extracts from agro-industrial food waste can be used as cross-linking agents in the modification of bovine gelatin. It has been demonstrated that the properties of gelatin can be adjusted to the desired level with a combination of the right extract type and concentration. Thus, a functional product with a wider range of applications can be achieved by modifying the qualities of gelatin that restrict its usage.

## Supporting Information Available

Light microscopic images of freeze-dried control and modified gels at different magnifications (Figure S1); variation of complex viscosity (η*) with frequency sweep (Figure S2); and Pearson correlation coefficients between all variables (Figure S3) (PDF)
