# Supplementary material for: Investigation of the Effects of Phenolic Extracts Obtained from Agro-Industrial Food Wastes on Gelatin Modification
Source: ACS Omega. 2024 Apr 24;9(18):20263–76. doi: 10.1021/acsomega.4c00690 (PMC11080024; doi:10.1021/acsomega.4c00690)
Supplement: Supplementary file 1 — ao4c00690_si_001.pdf [file ao4c00690_si_001.pdf]

Supporting Information

Investigation of the Effects of Phenolic Extracts Obtained from Agro-Industrial Food Wastes on Gelatin Modification

Huseyin Demircan[a,b*], Rasim A. Oral[a], Omer S. Toker[b], Ibrahim Palabiyik[c*]

[a] *Bursa Technical University, Faculty of Engineering and Natural Science, Department of Food Engineering, 16310, Bursa, Türkiye*

[b] *Yildiz Technical University, Faculty of Chemical and Metallurgical Engineering, Department of Food Engineering, 34210, Istanbul, Türkiye*

[c] *Tekirdağ Namık Kemal University, Faculty of Agriculture, Department of Food Engineering, 59030, Tekirdağ, Türkiye*

*Corresponding Authors: Huseyin Demircan (huseyin.demircan@btu.edu.tr) Ibrahim Palabiyik (ipalabiyik@nku.edu.tr)

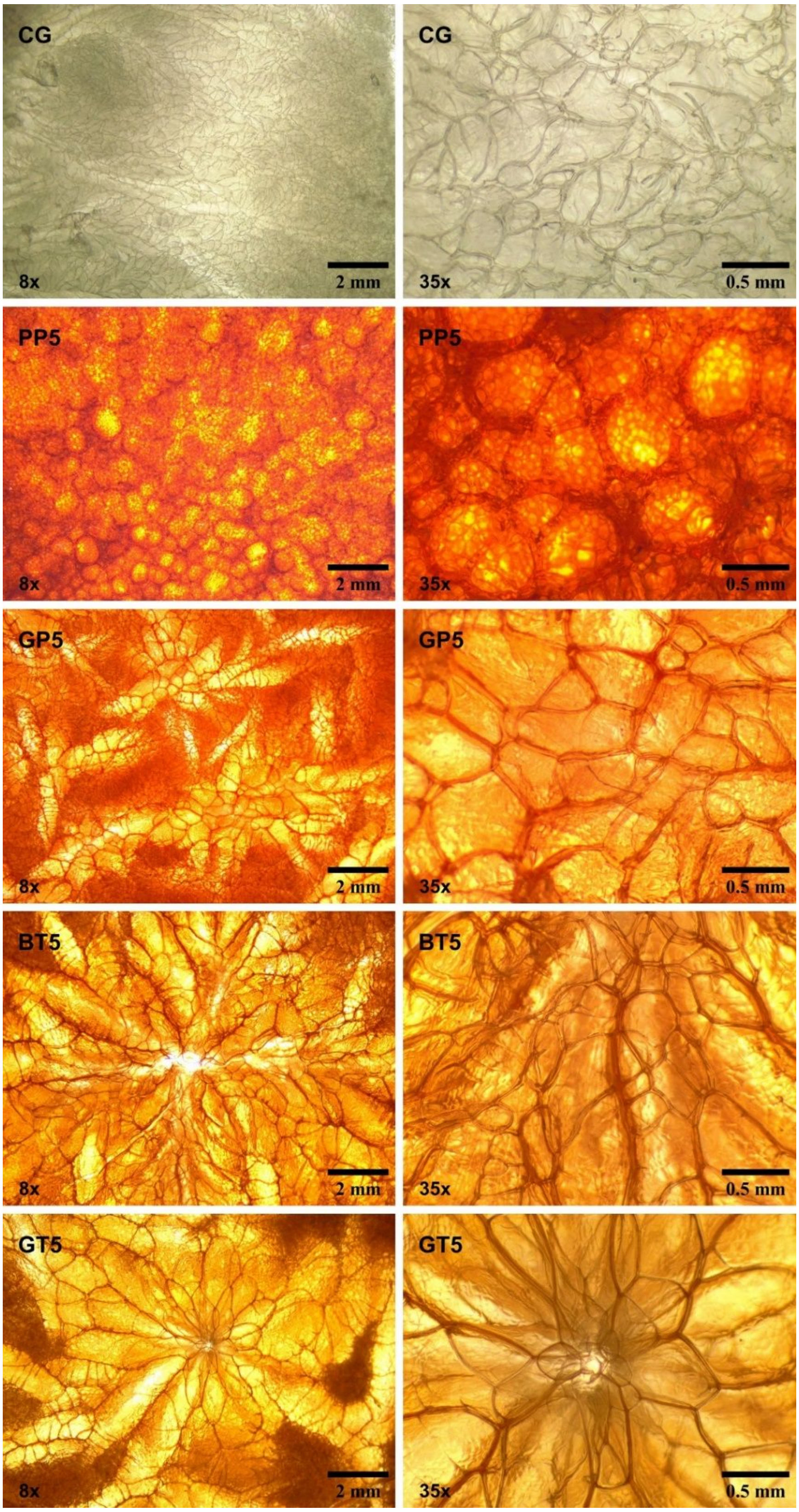

Figure S1. Light microscopic images of freeze-dried control and modified gels at 8x and 35x magnifications. CG: Control gelatin (without extract); PP: Gelatin crosslinked with pomegranate peel waste extract; GP: Gelatin crosslinked with grape pomace and seed waste extract; BT: Gelatin crosslinked with black tea waste extract; GT: Gelatin crosslinked with green tea waste extract; 5: Relevant extract ratio (%) used in the modification.

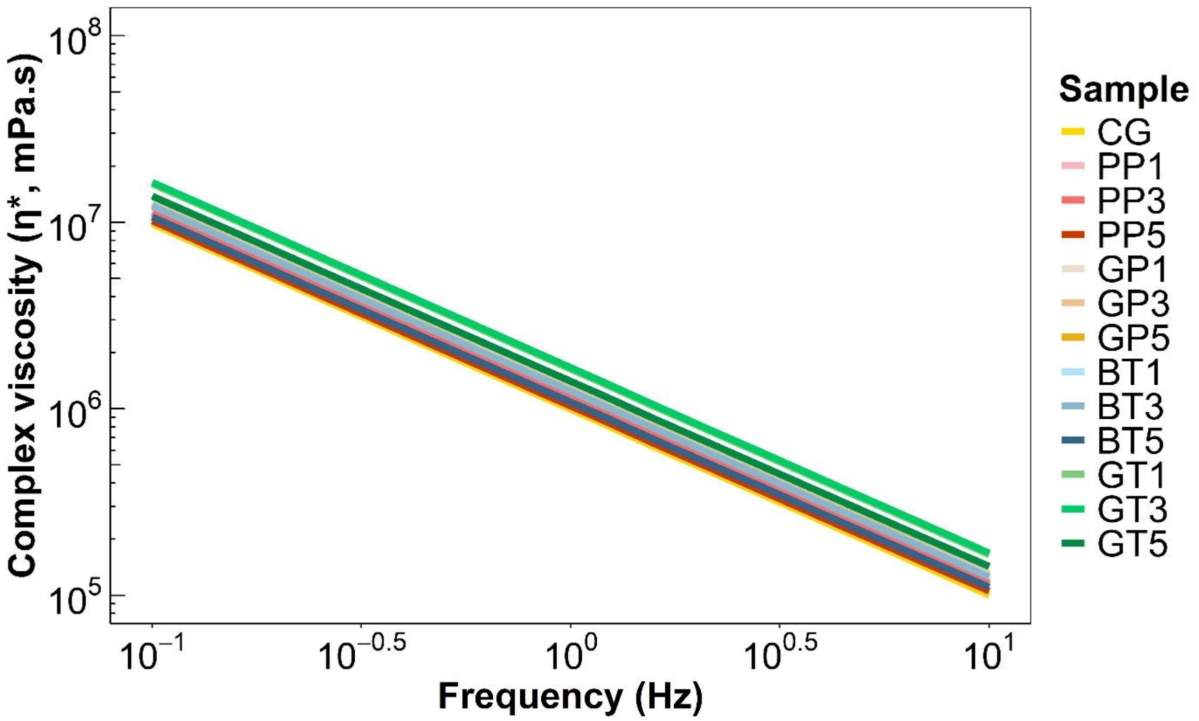

Figure S2. Variation of complex viscosity (η*) with frequency sweep. CG: Control gelatin (without extract); PP: Gelatin crosslinked with pomegranate peel waste extract; GP: Gelatin crosslinked with grape pomace and seed waste extract; BT: Gelatin crosslinked with black tea waste extract; GT: Gelatin crosslinked with green tea waste extract; 1-3-5; Relevant extract ratio (%) used in the modification.

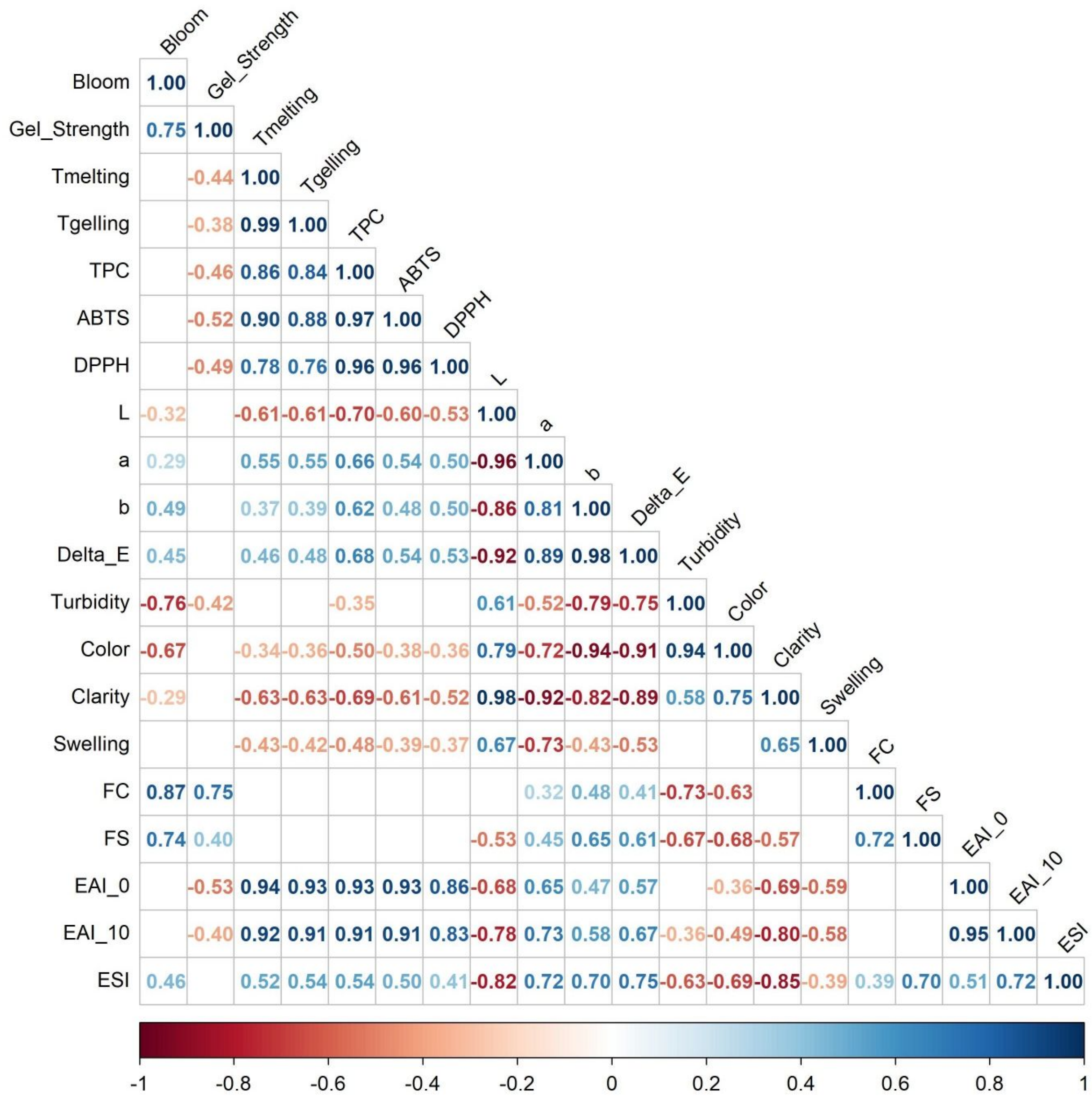

Figure S3. Pearson correlation coefficients between all variables (correlation values that were not statistically significant were excluded).
